# A mummified human corpse and associated insects of forensic importance in indoor conditions

**DOI:** 10.1007/s00414-020-02373-2

**Published:** 2020-07-14

**Authors:** Marcin Kadej, Łukasz Szleszkowski, Agata Thannhäuser, Tomasz Jurek

**Affiliations:** 1 Department of Forensic Biology and Entomology, Faculty of Biological Science, University of Wrocław, Przybyszewskiego 65, PL-51-148 Wrocław, Poland; 2 Department of Forensic Medicine, Wrocław Medical University, Mikulicza-Radeckiego 4, PL-50-345 Wrocław, Poland

**Keywords:** Decomposition, Forensic entomology, *Dermestes*, Insects, mummified human corpse, Indoor

## Abstract

We report, for the first time from Poland, the presence of *Dermestes haemorrhoidalis* (Coleoptera: Dermestidae) on a mummified human corpse found in a flat (Lower Silesia province, south-western Poland). Different life stages of *D. haemorrhoidalis* were gathered from the cadaver, and the signs of activity of these beetles (i.e. frass) were observed. On the basis of these facts, we concluded that the decedent, whose remains were discovered in the flat on 13 December 2018, died no later than the summer of 2018, with a strong probability that death occurred even earlier (2016 or 2017). A case history, autopsy findings, and entomological observations are provided. The presence of larvae of Dermestidae in the empty puparia of flies is reported for the first time. A list of the invertebrate species found in the corpse is provided, compared with available data, and briefly discussed.

## Introduction

Among a large number of experimental papers, all highly advanced in both methodical and technical terms are also papers describing specific cases relating to observations of insects on human remains accidentally discovered in extremely diverse places and conditions (natural vs anthropogenic; outdoors vs indoors), often after the passage of many months, or even years, after death [1–11]. The authors of these publications describe extremely diverse cases that certainly extend our knowledge in the field of forensic science. The paper we present here is part of this trend. It was written based on observations recorded by our team during the autopsy of the body of a man found in a block of flats in Wrocław in 2018.

The aim of this study was to describe and discuss first observations of *Dermestes haemorrhoidalis* (Coleoptera: Dermestidae) on a corpse found in a flat in Wrocław (Lower Silesia province, south-western Poland). In addition to the identification of this species, we describe the interesting behaviour of final-stage larvae associated with the use of empty casings of flies, probably as shelters for future pupae. We also discuss our other entomological findings and compare them with available data.

## Material and methods

**Fig. 1:**
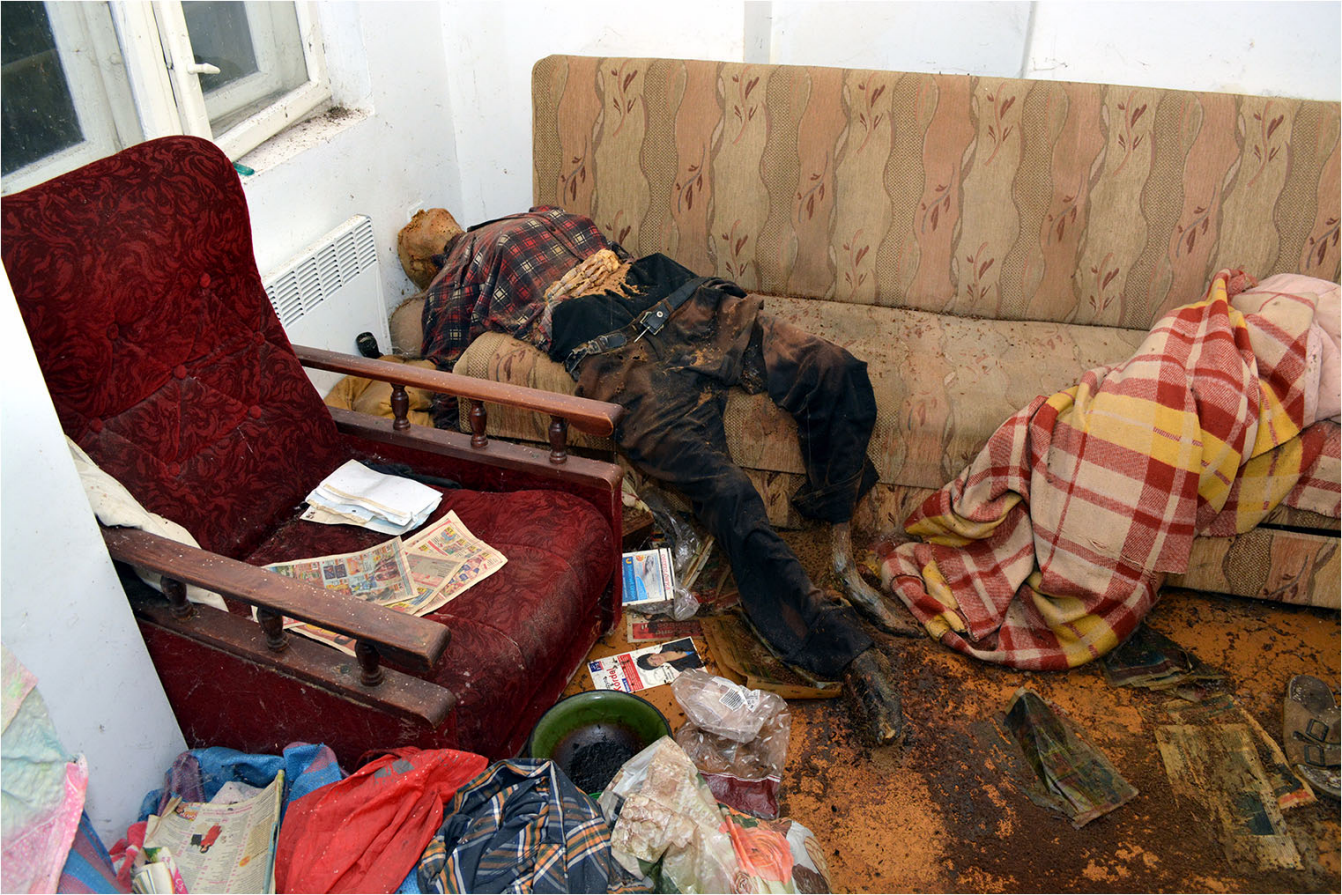
View of the remains at the site of their discovery (dorso-lateral view)

**Fig. 2:**
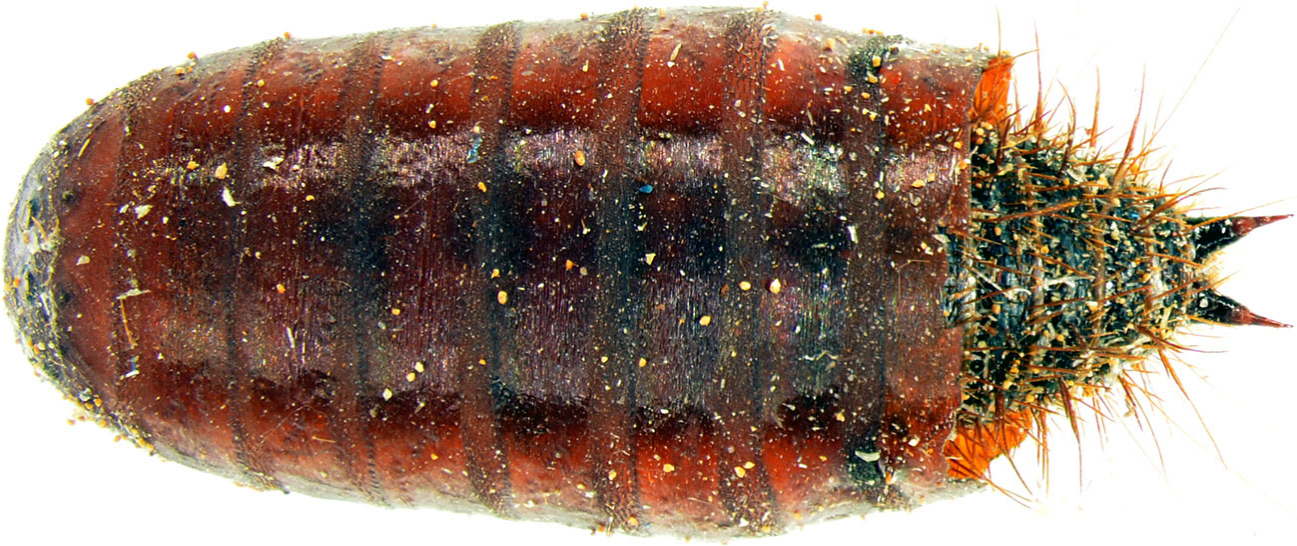
Larva of *Dermestes haemorrhoidalis* in the interior cavity of puparium of *Calliphora vicina* (general view)

**Fig. 3:**
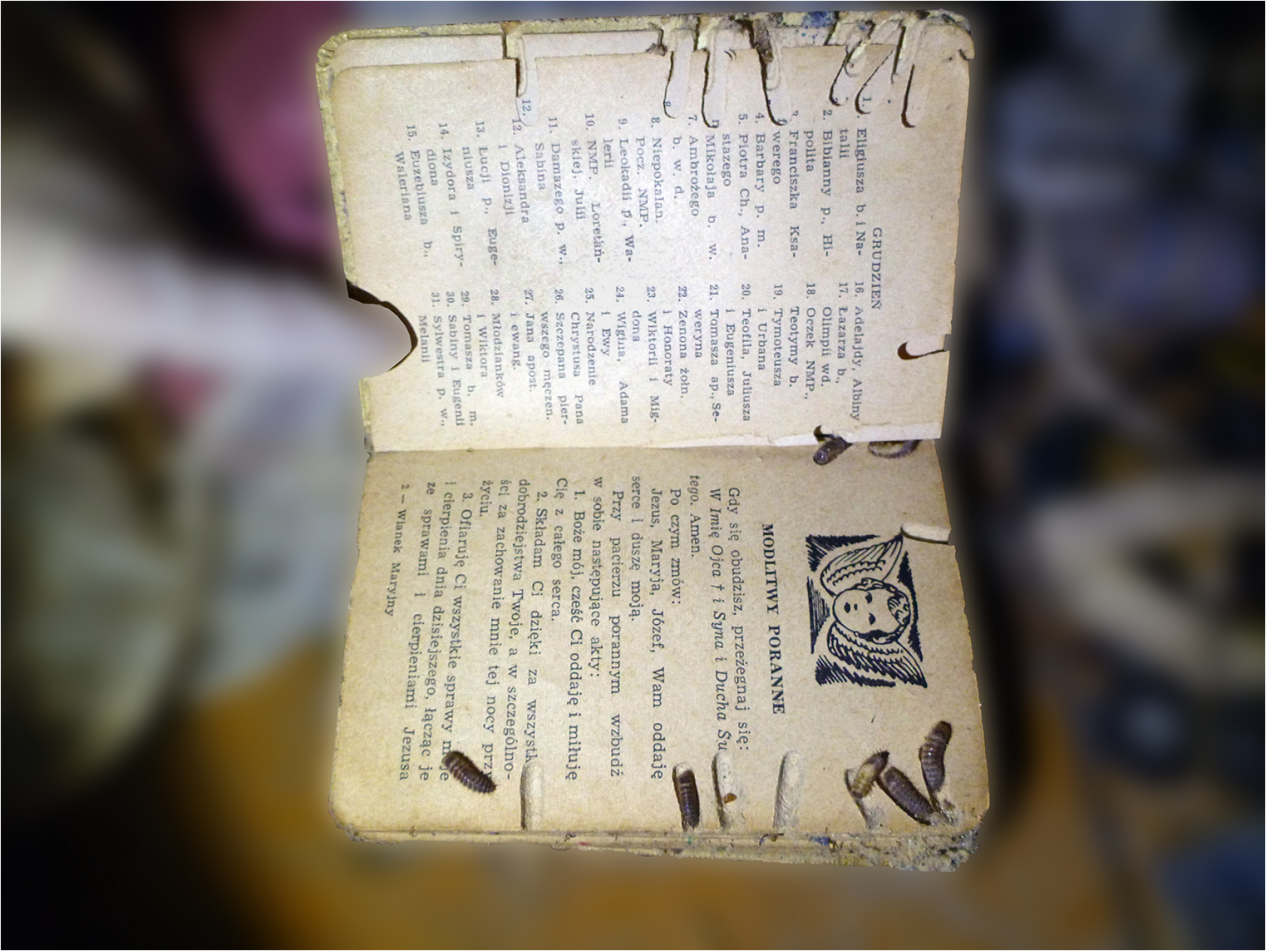
The interior of a prayer book with larva, larval tunnels and pupal chambers of *D. haemorrhoidalis* (general view)

**Fig. 4:**
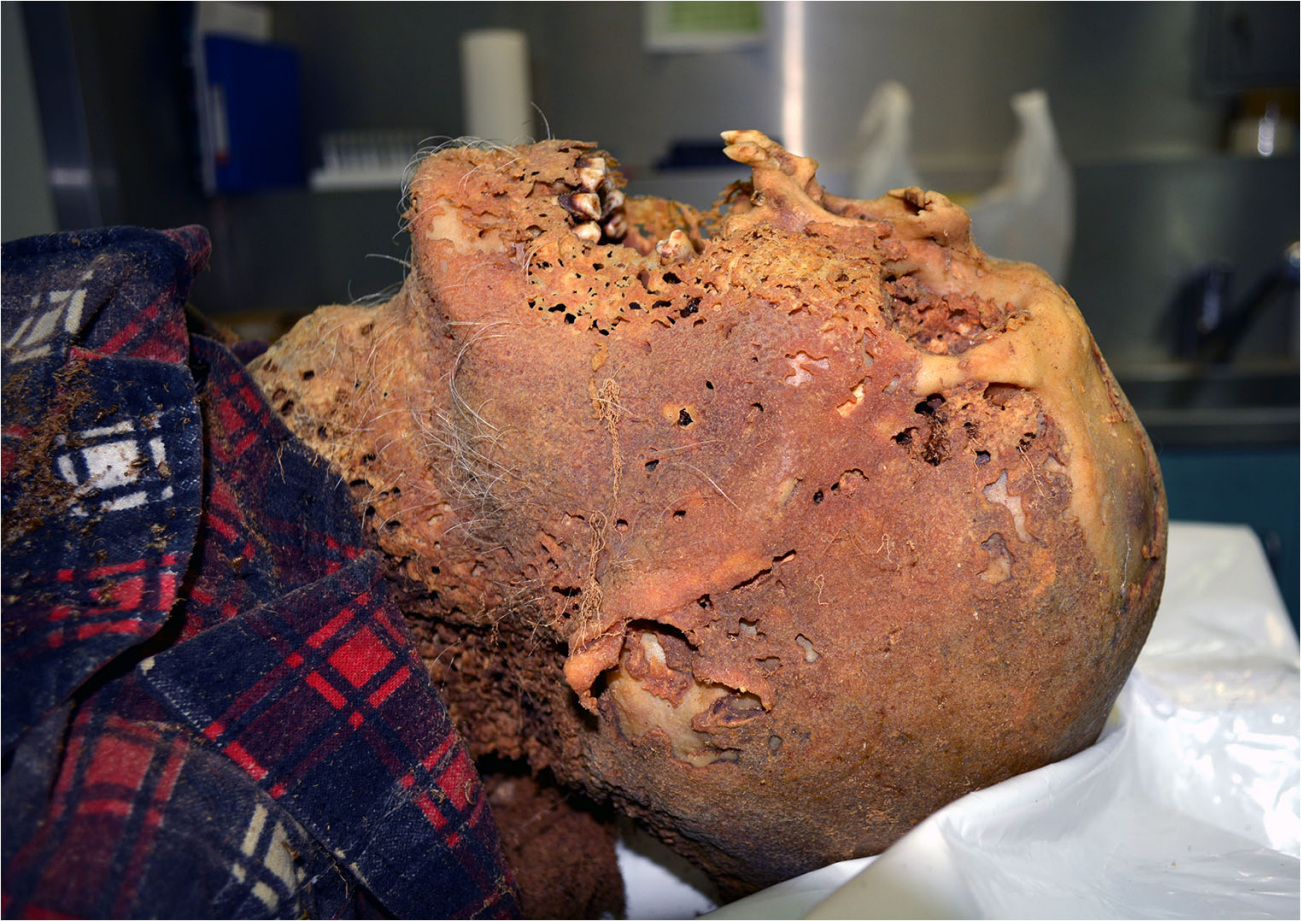
The openings in the mummified skin of the head due to the feeding activity of Dermestidae (general view)

**Fig. 5:**
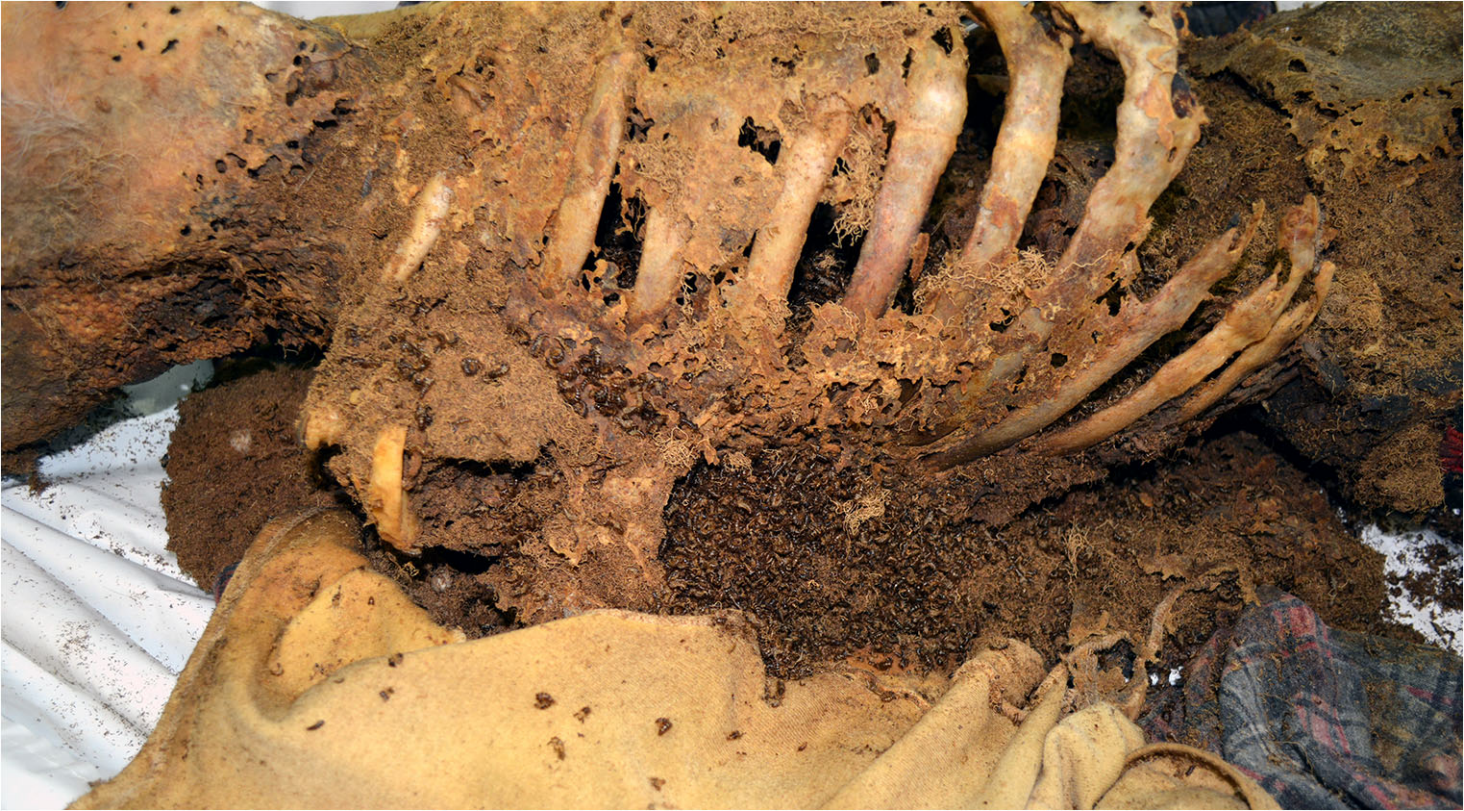
The feeding activity of Dermestidae on the chest (general view)

At the request of the prosecutor, a full standard forensic medical and anthropological examination and autopsy of the remains of the decedent were carried out. These activities were performed by a forensic specialist and forensic anthropologist with the participation of a forensic entomologist. Insects were stored in plastic vials for morphological examination. The studied material was collected directly from the corpse in the autopsy room and in the flat of the decedent as well. Species identification was carried out using a Nikon SMZ800 binocular microscope. Identification of spiders was verified by a specialist in arachnology, Dr. Robert Rozwałka (Poland). Photos (Figs. 1, 2, 3, 4, 5) were taken straight on with a Panasonic Lumix DMC-FT3 camera. A qualitative (not quantitative) analysis of entomological findings was conducted (Table 1).

**Table 1:** List of taxa found on the corpse and in the flat

Order/family/taxon	On/in the body (autopsy room)	Outside the body (adjacent to/under the body—autopsy room)	In the flat (i.e. on the couch, floor, carpet, blanket)
Coleoptera: Dermestidae *Dermestes* (*Dermestes*) *haemorrhoidalis* Küster, 1852	+ (larval skins, live larvae, dead and live adults)	+ (larval skins, live larvae, dead and live adults)	+ (larval skins, live larvae, dead and live adults)
Coleoptera: Dermestidae *Anthrenus* (*Nathrenus*) *verbasci* (L., 1767)	–	–	+ (live adult and larva reared to adult)
Coleoptera: Dermestidae *Attagenus smirnovi* Zhantiev, 1973	–	–	+ (live larvae reared to adults)
Diptera: Calliphoridae *Calliphora vicina* Robineau-Desvoidy, 1830	+ (empty puparia)	+ (empty puparia)	+ (dead adults, wings, and empty puparia)
Lepidoptera: Tineidae *Tineola bisselliella* (Hummel, 1823)	+ (dead adult and live larva reared to adult)	–	+ (dead adults)
Lepidoptera: Pyralidae unidentified species	–	–	+ (dead adults)
Araneae: Theridiidae *Steatoda grossa* (C.L. Koch, 1838)	+ (one live female, one juvenile specimen)	–	+ (live adults of both sexes)

## Results

### Scene of discovery of the remains

A 60-year-old man lived in a municipal flat located on the fourth floor of an old pre-war block of flats in the centre of the city, maintaining no contacts with his neighbours. On 13 July 2015, the tenant testified, in documents he signed himself, that he was familiar with the rate of payment for the flat. In January 2016, he failed to pick up a letter from the manager. From June 2016 to October 2018, the manager of the block of flats carried out four local inquiries (asking neighbours), which failed to establish the tenant’s presence in the flat. Interviewed by the police, neighbours were unable to confirm clearly and precisely the last time they had seen the tenant or when they had last heard sounds indicating his presence in the flat. According to the block’s residents, as of May 2018, no one had been living in the flat in question for at least a year. One neighbour stated that in the summer of 2018, ‘insects that had not been there previously’ had appeared in her flat. On 13 December 2018, the manager of the block entered the flat in question, discovering human remains therein. The police were immediately notified of this discovery. The corpse lay on its back at one end of a couch, at an angle oblique to its long axis (Fig. 1). The middle and lower parts of the back, buttocks, and parts of the thighs were lying on the couch, while the lower limbs and feet rested on the floor. On the floor in the vicinity of the decedent’s legs, as well as on the couch in the vicinity of the remains, were irregular dried brown stains from purge fluid, numerous larval instars, and dead adult specimens (chiefly beetles and flies) as well as accumulated frass. On 21 December 2018, a detailed forensic-entomological inspection of the site was carried out with the participation of an expert forensic entomologist and forensic anthropologist. During the inspection, samples with entomological material were secured from the room in which the decedent was found. Entomological traces were taken from furniture, floors, window sills, and objects found in the room (mainly on the floor) such as blankets, books, footwear, plastic bags, and clothing.

### Autopsy findings

The remains had been significantly skeletalised and subjected to subsequent fixative changes, i.e. transformed posthumously and dried (mummified), with residually preserved and transformed soft tissues. Present on the remains was clothing, which had dried out and adhered to skin integument. Fragments of advertising flyers from the Biedronka supermarket chain, dating from 2013, were dried and stuck to the clothing.

Skin integument was partly preserved on the head, neck, and anterior and lateral surfaces of the trunk, as well as on the back, buttocks, lower legs, feet, and right hand. On the face, neck, and chest, the skin integument had taken on a yellowish-brown colour and was hard, dry, greasy on the outer surface, matt, rough, and dried, adhering to the bones. On the face, a defect in tissue measuring 18 × 15 cm, with irregular margins, was present. Within the dried skin integument, numerous interconnected cavities were present: more or less regular, mostly round openings, with diameters of 0.1–0.5 cm and fairly even edges. On the surface of the chest, extensive loss of skin integument revealed the sternum, ribs, and collarbones. On the back, buttocks, and shins (posterior surfaces of the body), skin integument was matt, brown, and greasy on the outer surface; on the back, it was hard and dry, characterized by a texture corresponding to the weave of the decedent’s clothing; the edges of the preserved skin patches were uneven and jagged and on the feet was partially preserved, greyish, dry skin resembling parchment. Both femurs and the proximal part of the left lower leg were exposed. On the lower limbs, directly covering the bones, greyish-yellow patches of dry, greasy tissue were preserved, with irregular edges and numerous openings, usually round, with diameters of approximately 0.5 cm. Spinal ligaments, ribs, and pelvis were preserved in the form of dry, brown, greasy, hardened bands connecting bones and dried so as to adhere strongly to them; ligaments of the limbs were similarly preserved. Articular cartilage was pale brown, greasy, and dry. Internal organs were residually preserved but greatly transformed and characterized by a completely obscured morphological structure. Present in the skull cavity were dry, hard, brown patches of dura mater, greasy on the external surface, and the transformed brain, which was hard, compact, clayey, brittle, and blackish-brown, measuring 10 × 11 × 3 cm. Within brain, cavernous openings with diameters of approximately 0.5 cm were present, forming corridors several centimetres in length, in which was found frass of larvae and adult specimens of Dermestidae. In addition, loose frass was found preserved in the skull cavity along with numerous larval instars and dead adults of skin beetles. Blackish-brown, dry, greasy, slightly shrunken cartilage of the larynx and trachea was preserved in the neck area. Inside the chest, the remains of internal organs were preserved: the lobes of the lungs, pericardial sac, and diaphragm in the form of hard, dry material, greasy on the external surface, blackish-brown, resembling thick parchment or greasy cardboard, with numerous openings representing the consequences of beetles feeding on the body as described above. Also preserved were dark brown to brown, dried, greasy, and clay-like fragments of liver, characterized by a completely obscured morphological structure.

### Entomological findings

#### Invertebrates found on the corpse

During the autopsy, several puparia of *Calliphora vicina* (Diptera: Calliphoridae) were observed on the decedent’s clothing, along with dead beetles *Dermestes haemorrhoidalis* (Coleoptera: Dermestidae). These were present in much greater numbers inside the body, specifically in the pelvis, abdominal cavity, chest, throat, and cranial region. Numerous larval specimens of *D. haemorrhoidalis* were found in the chest, mouth, and skull cavity, representing various larval instars, with the final stages of development being predominant. Living larvae of *D. haemorrhoidalis* were found foraging in the skull cavity (2 specimens) and beneath the body of the decedent in the vicinity of the legs. Also present in the skull, in addition to larval instars and live larvae of skin beetles, were dead adult specimens of *D. haemorrhoidalis*, a live caterpillar, and a dead adult specimen of the moth *Tineola bisselliella* (Lepidoptera: Tineidae). In addition to insects in the body of the decedent, a live female and a juvenile spider *Steatoda grossa* (Araneae: Theridiidae) were also observed in the chest region*.*

#### Invertebrates found in the flat

Virtually everywhere in the decedent’s flat—on the floor, on the couch, in the neighbourhood of the couch, on the window sills and in cabinets, and on the blanket—were puparia of *Calliphora vicina* (Diptera: Calliphoridae), larval instars and dead adults of *Dermestes haemorrhoidalis* (Dermestidae), dead adults and living larvae (stadia L1, L2) of *Anthrenus verbasci* (Dermestidae), and living larvae (stadium L1) of *Attagenus smirnovi* (Dermestidae). In addition, a live larva of the moth *Tineola bisselliella* was found, as well as dead adult butterflies (Lepidoptera), probably from the families Tineidae and Pyralidae (identification to the species level was impossible due to the state of preservation of the collected and secured remains).

Represented in the greatest numbers were puparia of flies (Calliphoridae) as well as larval instars and adults of skin beetles (Dermestidae). Dead moths with dead larvae of *D. haemorrhoidalis* and one dead adult of *Calliphora vicina* were present in a dried stain of purge fluid approximately 1 m from the couch, where the decedent’s feet were located. Living larvae of skin beetles (several individuals) appeared between the folds of a blanket lying on the floor next to a pile of books and other small items. In addition, their presence was observed in the interiors of puparia (Fig. 2) lying on the floor near the blanket. Fourteen larvae of *D. haemorrhoidalis* were extricated from the interior of a prayer book, from which they had eaten out tunnels and pupal chambers (Fig. 3). These were mainly larvae in more advanced stages of development. In addition, in the course of the inspection, 3 larvae of *Attagenus smirnovi* (stadium L1) and one living larva of *Anthrenus verbasci* (stadium L1) were found. Among the objects accumulated on the floor, a live female and male of *Steatoda grossa* were observed.

#### Traces of invertebrate activity

Many openings were found in the decedent’s mummified skin due to the feeding activity of Dermestidae (Figs. 4, 5). The openings were present on the scalp, neck, chest (intercostal spaces), hands, and legs (fibular section), including the soles of the feet and the skin of the back. Traces of feeding larvae, probably including moths, were present on the decedent’s clothing—T-shirt and shirt—along with visible damage. In the body cavities (chest, abdominal cavity), beneath the underwear and in the immediate vicinity, frass was present as a result of the activity of foraging larvae of *D. haemorrhoidalis.* A total of 1734 g of frass was collected and secured on the dissection table.

The feeding activities of spiders *Steatoda grossa* present on the body were evidenced by numerous adults of *D. haemorrhoidalis* entangled in spider webs.

In the decedent’s flat, approximately 200 g of frass was collected from the couch and directly underneath it. Damage, consisting of shallow corridors and openings, was found in the upholstery of the couch. The corridors corresponded to the area in which the body of the decedent had been lying. In addition to damage to the couch and prayer book (Fig. 3), similar damage was observed in other items such as blankets and newspapers and a leather belt.

## Discussion

Found in the decedent’s remains, as well as in his flat, were empty puparia of flies from the family Calliphoridae. This flies are among the first insects to appear on corpses [12–15], often literally within a few minutes to hours from the moment of death [16], and focus on consuming soft tissues and internal organs [17]. Analysis of morphological features confirmed that they belonged to *Calliphora vicina.* This species, along with *C. vomitoria*, is among the species characterized by the most widely dispersed populations in temperate climates [16]. This species prefers shady locations and urban habitats, where it is often the dominant species on human cadavers [16, 18], and has sometimes been collected indoors [19]. This only confirms the legitimacy of its occurrence on the remains of a decedent in a flat in a block in the centre of a city. The development of calliphorids, including *C. vicina*, requires a substrate of suitable humidity [10]. Given that the remains must have been in the apartment for a period greater than several months, gradually succumbing to the process of mummification, the dried-out body was not a suitable habitat for the continued development of these flies. The presence of mummified remains of internal organs does not exclude the activity of Diptera maggots on the corpse, as evidenced by numerous puparia found in the deceased’s apartment. Rather, it proves that the apartment was in an extremely favourable condition for shortening the phase of active decomposition, thus forcing the fly larvae to withdraw from the corpse. Conditions in the flat during the inspection, on the other hand, were suitable for the development of second-order necrophages, such as skin beetles (Dermestidae) and fungus moths (Tineidae) [17]. Representatives of these two families feed mainly on remains such as dried-out skin and bones in the late stages of decay [12].

The sample of available publications concerning skin beetles in human corpses is relatively small. Cases of observation of larvae and adults or signs of the activity of these beetles on human corpses have been described by, inter alia, Voigt [1, 3–10, 16, 20–25]. To date, in cases of skin beetles of the genus *Dermestes* found in human remains, observations have involved species such as *Dermestes frischii* [8–10], *D.* (*D*.) *undulatus* [8, 10], *D. ater* [7, 8, 21], *D. bicolor* [8], *D. carnivorus* [21], *D. maculatus* [2, 4, 8, 11, 15, 22, 26], *D. lardarius* [1, 8, 23, 27], and *D. peruvianus* [8]. As shown by the analysis of 81 cases of observations of skin beetles in human remains, the species most frequently observed in France have been *D.* (*D*.) *frischii* and *D.* (*D*.) *undulatus* [8]. It is precisely these two species that have been most often recorded on cadavers in ‘outdoor’ conditions. This is confirmed as well by the work of other researchers [1, 9, 10]. The species most frequently recorded in the so-called ‘indoor’ conditions has been *D. peruvianus* [8]. Other species found in corpses in the same conditions (e.g. in flats) include *D. lardarius* [3, 23], *D. maculatus* [5, 22], *D. ater* [7], and *D. haemorrhoidalis* [3; the present paper]. In the case of Poland, skin beetles were found in experimental studies carried out on the body of a domestic pig (*Sus scrofa* f. *domestica*) [28–32] as well as in the corpse of a hanged man in natural conditions [1]. In the case of experimental research on the carcasses of pigs, the presence of species such as *D. frischii* [25, 28, 29, 32], *D. murinus murinus* [28], and *D. laniarius laniarius* [31] was found. *D. lardarius*, *D. frischii*, and *D.* (*D*.) *undulatus* appeared in the mummified corpse of a man in forest conditions [1]. In connection with the above, the observation of *D. haemorrhoidalis* in human remains must be acknowledged as the first in Poland.

With respect to representatives of the skin beetle family, our observations confirmed the presence of frass in both the decedent’s apartment and the remains. Frass is an obvious sign of foraging by skin beetle larvae [14–16, 20, 33–35]. These structures were also included in descriptions by, inter alia, Voigt [3] from Denmark; Schroeder et al. [5] from Germany; Arnaldos et al. [22] from Spain; and Bonacci et al. [10] from Italy. In our case, the total weight of frass collected from the body and clothing was 1734 g. The papers we have analysed [10, 36] indicated the presence of frass, but not its weight. Amounts of frass are difficult to quantify macroscopically; therefore, in our case, we measured its weight. The amount of frass can undoubtedly testify not only to the fact of the feeding activity of larvae but also to the intensity of this activity and the total number of larvae (a large amount of frass indicates a large number of larvae; a small amount, a small number of larvae).

An interesting observation concerned the presence of motionless larvae of the last stage (concealed) in empty fly casings. Heretofore, there have been no descriptions of larvae of skin beetles in puparia of Diptera. We have found no such reference in any paper referring to the activity of juvenile stages. The literature describes cases of interactions between *Dermestes* spp. and flies [37, 38]. In one of them, researchers conducting an experimental study proved that *D. ater*, although a typical necrophage [12], exhibits predatory behaviour [37] in relation to larvae of *Musca domestica* (Diptera: Muscidae). Probably larvae of *D. haemorrhoidalis*, in the case we have described, behaved in the same way in preying on the pupae of blowflies in the decedent’s flat. It appears that our observation is the first of its kind to describe predatory behaviour on the part of larvae of *D. haemorrhoidalis* in relation to the pupae of flies. It cannot be ruled out that, following a meal, skin beetle larvae used the empty shells of pupae as a place for their own pupae. Interestingly, the larvae in the shells of fly pupae were found on the floor of the flat, not directly in or on the remains of the decedent. It is generally known that final-stage larvae seek places for the process of metamorphosis. These places are not necessarily associated with the larvae’s previous feeding activity. For the period of metamorphosis, larvae frequently move to secluded places, where they usually also gnaw out pupal chambers [14, 25, 39]. Pupal chambers created by *Dermestes* spp. have also been observed on human bones from the Middle Bronze Age [40] and in fossils from the late Pliocene and middle-late Pleistocene [41]. In our case, such pupal chambers were created in, inter alia, a prayer book and perhaps also in the couch on which the body of the decedent lay, as may be confirmed by the numerous openings in the upholstery.

As in the case described by Bonacci et al. [10], feeding damage caused by *D. haemorrhoidalis* specimens was present on the decedent’s skin. It cannot be ruled out that similar damage was also caused in the early stages of decomposition by fly larvae [16]. Although Schroeder et al. [5] reported a typical ‘larva-shaped’ feeding defect on the humerus of a cadaver discovered in an apartment 5 months after death, we did not observe any damage of this kind to bones.

In addition to *D. haemorrhoidalis*, two other species of skin beetles were present in the decedent’s flat, namely, *Attagenus smirnovi* and *Anthrenus verbasci*. Both species are characterized by a wide distribution at present and are among the species of the skin beetle family most commonly occurring in flats [42]. They are regarded as eminently synanthropic species which nourish themselves on various food sources in flats. It should be emphasized that no representatives of *A. verbasci* or *A. smirnovi* were observed directly on the remains of the decedent.

Spiders, as specialized predators, occur wherever their potential victims, i.e. insects, are found. Thus their presence in the decedent’s apartment, in which adults of both sexes of *Steatoda grossa* were found, is not surprising. In the webs, the spiders’ main victims were the same insects found in the body of the decedent (most often adult specimens of *D. haemorrhoidalis* and flies). In the area of the chest, we observed a live female and a juvenile specimen of *Steatoda grossa* and dead specimens of adult skin beetles entangled in the web.

## Conclusions

Skin beetles, especially those in the genus *Dermestes*, can be of considerable forensic importance [7, 10, 12, 16, 22, 25, 42–47]. Among closely related *Dermestes* species, it is currently assumed that only a few are regarded as indicators of minPMI. According to Magni et al. [25], species of forensic relevance also include taxa such as *D. maculatus*, *D. lardarius*, *D. ater*, and *D*. *frischii*. Missing from this list is *D. haemorrhoidalis*; however, due to its close kinship with the aforementioned species, it can be assumed that this species has a similar significance for forensic entomology. Obviously, in order to confirm this, further work is needed to document the arrival and oviposition timeframes of this species in conditions prevailing at death scenes.

Dermestid beetles are considered very late colonizers, frequently arriving when only skin and bone remains, sometimes months after death [12, 48–50]. In some cases of mummification, living dermestid adults and larvae may still be associated with the remains after a period of years [3, 12, 51]. In our case, based on the presence of so many dead adults and larvae, and especially of larval instars and frass, it can be unequivocally acknowledged that a relatively long time (even years) must have passed between death and discovery of the remains. Numerous larval instars, dead adults, and living larvae (found within the skull) are proof that probably as many as several generations of these beetles may have developed in the remains prior to their discovery. Our inference results from scientific premises indicating that even though skin beetles appear on corpses within 10 to 13 days, i.e. during the period of active decay [14, 35, 43, 52, 53], they most often occur on exposed remains in a temperate climate during the third wave of decomposition, when the fats are rancid (after 3–6 months) [25]. It has also been indicated that other species of the family Dermestidae are most prevalent during the seventh wave, when the remains are completely dry (after 1–3 years) [25]. The development of *D. haemorrhoidalis* from egg to mature adulthood takes an average of 145 days [39]. The thesis positing a considerable interval between death and discovery is also supported by the observation of the moth *Tineola bisselliella*. This species usually appears on the corpse in the seventh stage of arthropod succession, i.e. when the remains are completely dry [12, 22]. On the basis of these facts and taking into account the biology of the *D. haemmorhoidalis*, we concluded that the decedent, whose remains were discovered in the flat on 13 December 2018, died no later than the summer of 2018, with a strong probability that death occurred even earlier (2016 or 2017), as possibly indicated, inter alia, by the significant number of larval instars and by the damage to the surfaces of some of the collected empty fly puparia.

In conclusion, all cases enabling observation of the succession of insects on human remains occurring under natural conditions (in nature) and in anthropogenic environments are of great value for a better understanding of the processes associated with decay. Observations of individual species on human corpses not only supplement our current state of knowledge about their biology and ecology but also often supply completely new facts which may then prove useful in forensic entomology, in the widest understanding of the term.
